# Variation of rabbit serum IgA in relation to RHDV vaccination and infection: implications for serological diagnosis and epidemiological investigations

**DOI:** 10.1186/s13567-026-01774-2

**Published:** 2026-06-09

**Authors:** Lorenzo Capucci, Vittoria Di Giovanni, Michele Schiavitto, Patrizia Cavadini, Antonio Lavazza, Brian Cooke

**Affiliations:** 1 WOAH Reference Laboratory for Rabbit Haemorrhagic Disease, Istituto Zooprofilattico Sperimentale della Lombardia e dell’Emilia-Romagna “Bruno Ubertini” (IZSLER), 25124 Brescia, Italy; 2 Italian Rabbit Breeders Association (ANCI-AIA), 71030 Volturara Appula, FG Italy; 3 Rabbit-Free Australia, Collinswood, Adelaide, SA 5081 Australia

**Keywords:** Rabbit, rabbit haemorrhagic disease, serology, ELISA, vaccine, IgA, oral challenge, subcutaneous challenge

## Abstract

Rabbit haemorrhagic disease (RHD) is a highly pathogenic and contagious disease caused by RHD virus (RHDV) and RHDV type 2 (RHDV2), two distinct *Lagovirus europaeus* belonging to the *Caliciviridae* family. Serological investigations have been widely and successfully used on both wild and farmed rabbit populations. However, interpreting serological data remains uncertain, especially for IgA results in previously infected or vaccinated rabbits. The first part of this study reports the results of two independent vaccination experiments that demonstrated (a) serum IgA was present only in trace amounts and for a few weeks in about half of the vaccinated rabbits and (b) the serological profile of vaccinated rabbits changed significantly, with a marked and rapid increase in IgA, only when they were challenged with live RHDV administered orally. The second part of the study was carried out on a farm over 3 months, during which an RHDV outbreak unexpectedly occurred. We repeatedly sampled nine multivaccinated does, finding constant and medium-to-high levels of IgA in only three of them, namely the oldest ones, which had already been present a year and a half earlier during a devastating RHDV2 epidemic. At least one of the does became IgA positive, coinciding with the RHDV outbreak. A parallel survey on 61 young rabbits living in separate sheds on the farm showed a high percentage of IgA-positive rabbits in just one shed. Overall, our findings definitively demonstrate that vaccinations do not induce serum IgA, except in previously infected rabbits, and that IgA can be used as a marker of RHDV reinfection.

## Introduction

Rabbit haemorrhagic disease (RHD), a deadly form of hepatitis, is a serious disease in lagomorphs, with both high morbidity (nearly 100%) and a mortality rate (over 80%) [1, 2].

RHD is widespread globally, affecting the European rabbit (*Oryctolagus cuniculus*), whether wild, farmed or kept as a pet. RHD is caused by two related *Lagovirus europaeus* within the *Lagovirus* genus, *Caliciviridae* family [3]: the RHD virus (RHDV, genotype GI.1), first described in 1984 in China [4], and the RHDV type 2 (RHDV2, genotype GI.2), which emerged around 2010 in Europe [5–7] and largely replaced the original RHDV worldwide within a few years. Despite their notable genetic similarity, they have distinct antigenic profiles [6]. RHDV2, unlike RHDV, can also cause RHD in rabbits less than 2 months old [5] and may also affect many other lagomorph species (*Lepus* spp. and *Sylvilagus* spp.), which are currently considered secondary hosts [8], i.e., affected by RHD only if there are simultaneous outbreaks of RHD in European rabbits living nearby.

The oral/nasal transmission route of RHDV appears to be the most common in farmed rabbits, with the intestinal mucosa acting as the primary site of replication. However, it has been suggested that insect bites or, more often, the ingestion of food contaminated with infective fly-spot (faeces or regurgitate) from flies that have fed on dead rabbit carcasses, may be significant transmission routes in the field for wild rabbits [9, 10]. In any case, regardless of the route of infection, the virus quickly reaches the liver via the bloodstream, where it replicates extensively, particularly in hepatocytes, leading to lethal hepatitis within 42–72 h [11]. Interestingly, among the few rabbits that survived RHD, about 15% of a stock of 100 rabbits used for vaccine production with oronasal infection began producing specific immunoglobulins, IgM and IgA, as early as 72–84 h after infection (Capucci L. & Lavazza A., personal observations). Most of these rabbits recovered fully within a few days, developing long-lasting IgG titres that protected them from RHD for life. Moreover, it has been demonstrated that even very low levels of anti-RHDV IgG in serum can confer protection against RHD, in both convalescent rabbits and naïve rabbits inoculated with hyperimmune serum or anti-RHDV IgG [12–16]. This underpins the success of RHDV vaccines, which, regardless of their production method, when properly administered and managed on rabbit farms, have proven to be highly effective against RHD. In contrast to IgG, IgA disappears from the serum a few months after recovery from RHD, and the mucosal protection presumably diminishes as well [17]. Based on general principles of mucosal immunology, it is assumed that protection at the mucosal surface also declines as secretory IgA (sIgA) output decreases due to turnover [18, 19]. In the case of enteroviruses, primary IgA production occurs in response to the viral infection of intestinal epithelial cells. The process by which naïve B lymphocytes are activated and switch to IgA production is mediated by local associated lymphoid tissues [20–22].

Serological methods have played a key role in understanding RHD and developing strategies for managing rabbit populations. Since an effective in vitro system to replicate RHDV has only recently been reported [23, 24], initial serological tests relied on the virus’s ability to agglutinate human red blood cells (HA inhibition test) rather than traditional serum neutralisation methods [12, 25]. However, more straightforward, more robust and reliable enzyme-linked immunosorbent assay (ELISA) tests were soon developed and used [12, 17, 26]. Additionally, early serological studies using ELISA identified rabbits that tested positive for anti-RHDV antibodies despite never having been infected with RHD or vaccinated [12, 26–28]. These findings prompted studies that led to the discovery of several genetically and antigenically related rabbit caliciviruses (RCVs) (genotypes GI.3–GI.4), most of which are entirely non-pathogenic lagoviruses with intestinal tropism [29–33], that were hidden beneath the visible surface of the pathogenic RHDVs. Given the widespread occurrence of RCVs, serological diagnosis should always be considered to detect their possible presence in wild and farmed rabbit populations.

Following the emergence of RHDV2, serological ELISA methods were updated, and when used, they revealed the presence of virus-specific antibody subpopulations in sera. This is due to the consistent antigenic difference between the surfaces of the two viruses, which are nearly two distinct serotypes [12, 34, 35]. This enabled, on many occasions, the determination of whether RHDV or RHDV2 was circulating in a specific area at a particular time. This is especially useful in populations with a high proportion of seropositive rabbits and consequently rare cases of RHD [36].

Since the 1990s, vaccines have been developed to protect domestic rabbits from RHD. Consequently, there has been particular interest in whether specific IgA antibodies can serve as a useful marker to distinguish infected from vaccinated rabbits. At the same time, in Australia, where RHDV has been used as a biological control agent to reduce the abundance of wild rabbits, widely regarded as pests, researchers attempted to interpret serological data obtained from epidemiological surveys. In particular, they explored whether IgA positivity could indicate not only RHD convalescence but also repeated reinfection in rabbits protected from RHD by systemic immunity [17].

Based on these considerations, this study aimed to understand the factors underlying the variability in serum IgA responses to different immunogenic stimuli (infection or vaccination) and the rabbits’ status (naïve or previously infected/vaccinated). This was achieved by considering that the rabbit, such as other lagomorphs, has a highly complex IgA system, with at least 11 IgA subclasses expressed differently in the gastrointestinal tract [37]. Most differences between IgA isotypes relate to the length and amino acid composition of the hinge region, which explains the varying resistance to the numerous proteases acting in the intestine [38, 39]. In this context, we conducted independent experimental trials in Italy and Australia and used a serological approach to study two different scenarios: (a) wild and domestic rabbits that were experimentally vaccinated and then challenged with RHDV via different routes of exposure, either oral or by intramuscular/subdermal inoculation, and (b) farmed rabbits, investigating the changes in the serological profile over time, before and after a natural outbreak of RHDVa [40]. The results deepen our understanding of the roles of various Ig isotypes, especially IgA, in response to RHDV infection and vaccination.

## Materials and methods

### Experiments conducted in Australia

#### Animals

Ten laboratory-bred wild-type rabbits, aged 6–12 months, were housed individually in cages within an air-conditioned animal room at 18 °C (± 2 °C). These rabbits originated from animals collected several years earlier in the Canberra district and the southern tablelands of New South Wales.

#### Vaccination and sampling

Blood samples of about 1 mL were collected from a marginal ear vein of each rabbit 1 week before the experiment started to confirm that the rabbits were seronegative. Samples were then taken immediately before subcutaneous vaccination with a 1 mL dose of vaccine containing inactivated RHDV (Cylap HVD Vaccine, Cyanamid Websters Pty Ltd, Australia), adjuvanted with white mineral oil. Subsequently, blood samples were collected weekly after vaccination. Serum was extracted from each and stored at −20 °C until testing.

#### First RHDV challenge

The day rabbits were vaccinated was considered Day 0 of the experiment. At Day 49 (7 weeks after vaccination), blood samples were taken from all ten rabbits before they were challenged with 0.5 mL of liver homogenate containing 1500 median lethal dose (LD_50_) RHDV [Commonwealth Scientific and Industrial Research Organisation (CSIRO), Division of Wildlife and Ecology, Batch: RCV-1A]. Five rabbits were infected orally, and five were inoculated subcutaneously on the back between the shoulders. Serum samples were collected subsequently at weekly intervals.

The virus’s viability was confirmed through a concurrent experiment in which 12 domestic rabbits were orally inoculated with the same batch of virus (B. Cooke, unpublished data). All those rabbits developed acute RHD.

#### Second RHDV challenge

At Day 104, approximately 8 weeks after the first RHDV challenge (RHDV#1), a second challenge (RHDV#2) was conducted as described above. However, instead of simply reversing the treatments of each group of five rabbits (i.e. orally dosing the previously subcutaneously inoculated rabbits and vice versa), we randomly selected two new groups of five rabbits from the original ten rabbits, irrespective of previous treatment. This was done because we wished to know whether serological responses attributable to the route of inoculation would still be apparent even among rabbits with different histories of exposure to the virus. Serum from these rabbits was again collected weekly thereafter for assay and regular weekly blood sampling continued for 7 weeks after the second RHDV challenge.

Although not directly related to the aim of experiments outlined above, it was recognised that, during our work, two rabbits had received oral dosing in two consecutive treatments, three rabbits were given oral and then subcutaneous inoculation, three received subcutaneous then oral treatment, and the two rabbits in the final group were subjected to successive subcutaneous inoculations. As there were only two or three rabbits in each group, this precluded any statistical analysis; nevertheless, the data from individual rabbits was enough for visual comparisons, which helped us determine the scale of changes in IgA and IgG likely to be indicative of re-exposure to RHDV among wild rabbits caught in the field.

#### Statistical analyses

Where appropriate, reciprocal antibody titres were log-transformed for statistical procedures [analysis of variance (ANOVA), analysis of covariance (ANCOVA)], and the more stringent Tukey honestly significant difference (HSD) test was used instead of standard *t*-tests when multiple comparisons of successive data sets were made. This was conducted using Systat software (Systat Inc., Evanston, Illinois, USA). Standard deviations were employed to describe variability around the mean.

### Experiments conducted in Italy

#### RHDV strain used for the challenge

The homogenate used for the challenge was prepared from the liver of a rabbit infected with the RHDVBs89 reference strain that died of acute RHD 42 h after oral infection. This liver, stored in fragments at −80 °C, has been used in several infection experiments, demonstrating that it causes RHD in almost 100% of rabbits within 96 h of infection [41]. Using an Ultraturrax (IKA-Werke GmbH, Staufen, Deutschland) in 30 mM phosphate, NaCl 300 mM, ethylenediaminetetraacetic acid (EDTA) 1 mM, pH 7.2 at 20% weight/volume (w/v), 5 g of liver were homogenised. The raw homogenate was clarified by centrifuging twice at 12 000 *g* for 10 min, and the resulting supernatant was divided into two aliquots. All the preparatory steps described above were performed at 4 °C. One aliquot was diluted with an equal volume of glycerol and stored at −20 °C. Formalin was added to the other aliquot to a final concentration of 1%, left in agitation at room temperature for 4 h and then stored at 4 °C overnight. Finally, the formalin-treated homogenate was also diluted with an equal volume of glycerol and stored at −20 °C. Considering that we prepared a 10% w/v homogenate, which was further diluted 1:20 for oral administration, we used approximately 5 mg of tissue per rabbit. This corresponds to 10^8^–10^10^ RHDV genome copies, based on general literature data on acute cases of RHD. To test the effectiveness of the formalin treatment, three 3-month-old New Zealand rabbits, seronegative for RHDV and RCV, were orally inoculated with 1.0 mL of inactivated RHDVBs89 homogenate, diluted 1:20 in pH 7.4 saline-phosphate buffer. The rabbits were then continuously monitored for health status over 15 days. At the end of the period, a blood sample was collected from each rabbit for serological testing.

#### Rabbit vaccination and challenge

Six 3-month-old New Zealand rabbits, seronegative for RHDV and RCV, were subcutaneously vaccinated with Izovac Mevax 2 (IZO S.r.l., Brescia, Italy), a bivalent vaccine against RHDV (strain BS/89, 1:2048 HA/dose) and RHDVa (strain MI/5503, 1:2048 HA/dose) with aluminium hydroxide as an adjuvant. After vaccination, the rabbits were housed in single cages with ad libitum access to water and feed in a room within the biosecurity level 3 (BSL-3) animal facility, where they were continuously monitored for their health status.

Unfortunately, one rabbit died a few days after vaccination due to other causes, without showing any signs referable to RHD. At 65 days post-vaccination, rabbits were orally challenged with 1.5 mL of a liver homogenate containing RHDVBs89. Three and two rabbits, randomly selected, were orally administered the live and the formalin-inactivated virus, respectively. The two groups of experimental rabbits were housed in individual cages but moved to separate rooms. Rabbits were continuously monitored, and blood samples were collected from vaccination day (day 0) on days 9, 16, 34, 57, 65 (day of challenge), 74, 83, 95 and 130. The serum samples were stored at −20 °C until ELISA tests were conducted.

#### Serological survey in the farm

The farm involved in the study, located in the province of Foggia in southern Italy, belongs to the National Association of Italian Rabbit Breeders (ANCI), an organisation dedicated to the genetic improvement of Italian rabbit breeds. It is divided into ten separate sheds, each managed independently and accommodating up to 432 breeding and 288 replacement females. In Shed #3, only males (up to 500) are kept. Following a major outbreak of RHDV2 in March 2013, a strict hygiene and sanitation programme was implemented, on the basis of biosecurity measures, cleaning and disinfection routines and parasite control, as well as a vaccination scheme for both RHD and myxomatosis. Additionally, breeding and replacement females and males were periodically tested serologically to assess the effectiveness of vaccination. Despite the strict prophylactic measures, which clearly only partially limited the risk of pathogen introduction, given the frequent movements in and out of the farm (animals sold, feed, removal of carcasses, visitors, etc.), a new outbreak of RHD involving RHDVa occurred in August 2014. At that time, the entire farm housed 1883 breeding females, 107 breeding males, 1300 replacement females (approximately 90–120 days old), 9000 fattening rabbits younger than 55 days and 350 fattening rabbits older than 55 days. Essentially, the replacement females were vaccinated against myxomatosis and RHD at 35–40 days of age and then boosted 1 month later and subsequently every 4 months, as was the case for all females and males involved in production. The last vaccination was administered subcutaneously about 2.5 months before the RHD outbreak, using both an RHDV2 autovaccine produced by IZSLER and a bivalent commercial vaccine containing RHDV and RHDVa strains (Izovac Mevax 2, IZO S.r.l., Brescia, Italy). “Cunivax Myxoma” (Fatro Industria Farmaceutica Veterinaria S.p.A., Ozzano Emilia (BO, Italy) was used for myxomatosis vaccination.

Interestingly, mortality due to RHDVa was confined to shed #7 and affected only unvaccinated fattening rabbits more than 55 days old. Immediately after the outbreak, all rabbits on the farm, including those that were not vaccinated, received emergency vaccination with both the RHDV2 autovaccine and the bivalent RHDV/RHDVa vaccine (Izovac Mevax 2, IZO S.r.l., Brescia, Italy) to induce immunity against the homologous strain responsible for the outbreak. To note that emergency vaccination during an outbreak is a common practice in rabbit farms to reduce mortality and control the disease, especially when performed immediately after diagnostic confirmation. Since only rabbits kept as breeders are typically vaccinated preventively, vaccinating fattening rabbits can induce protective immunity within 4–7 days, resulting in survival among most individuals that would otherwise die, while reducing viral circulation and the duration of the outbreak.

##### Survey of multi-vaccinated females

Blood was collected weekly from 25 July 2014 to 25 September 2014 from female breeding rabbits. A total of nine rabbits were included in the sampling protocol: four rabbits were sampled only during the first 3 weeks, and five rabbits throughout the entire period.

##### Survey of young rabbits

Within the same period, 61 young rabbits of varying ages, born from females vaccinated as described above, were randomly selected from four different sheds for blood sampling. Of the rabbits, 36 were not vaccinated, while 25 were fully vaccinated (RHDV2/RHDV/RHDVa) after the outbreak began, as part of emergency measures to protect the rabbits and curb the spread of the infection.

### ELISA assays used in the survey

All the ELISA methods used have been widely employed in previous studies and described in various articles [17, 25, 34], so a brief overview of both competition ELISAs (cELISAs) and isotype ELISAs (IsoELISAs) follows.

#### Competition ELISA (cELISA)

The core of the cELISA is the competition that occurs during the first hour of plate incubation. This involves the rabbit IgG from a hyperimmune anti-RHDV or RHDV2 serum, which is adsorbed onto the ELISA plate, competing with the immunoglobulins in the test serum for the antigen (RHDV or RHDV2). The test serum is diluted between 1/10 and 1/10240, typically in a series such as 1/10, 1/40, 1/160 and so on, while the antigen, which is the limiting reagent, is diluted to achieve an optical density (OD) around 1.2. In the second step of the ELISA, the amount of bound antigen is semi-quantified using a specific anti-RHDV or RHDV2 horseradish peroxidase (HRP)-conjugated monoclonal antibody (MAb). Results are classified as negative, inconclusive (doubtful) or positive on the basis of the percentage of OD compared with the negative control serum at 1/10 dilution. If the percentage is over 85% (indicating no competition), the result is negative; between 85 and 75%, it is inconclusive; and below 75%, it is positive. Each positive serum titre corresponds to the dilution at which the competition percentage falls between 40% and 60%.

#### Isotype ELISA (IsoELISA)

Unlike cELISAs, in which all isotypes can contribute to the competition, iso-ELISAs detect only specific IgM, IgG or IgA against RHDV or RHDV2. The IgG ELISA is a classic direct ELISA reaction in which the antigen (RHDV or RHDV2) is bound to the solid phase via a specific MAb. The test serum is diluted four to six times from 1/40 in base 4 (1/40, 1/160, 1/640, 1/2560 and so on), and the IgG bound to the antigen is semi-quantified using a specific anti-rabbit IgG HRP-conjugated MAb. Since IgG is the predominantly expressed isotype in serum, to obtain iso-ELISAs with the highest possible analytical sensitivity, the ELISA methods for IgM and IgA are inverse ELISAs. In both ELISAs, the specific isotype (IgM or IgA) is captured from the serum and bound to the solid phase via a specific pre-adsorbed anti-isotype MAb, which specifically recognises the H chain of immunoglobulins and has been used in several previous studies [17, 28, 42, 43]. Again, test sera are diluted to 1/40, and then in base 4. Then, the antigen (RHDV or RHDV2) is incubated under saturating conditions, and finally, an HRP-conjugated specific MAb (anti-RHDV or anti-RHDV2) is used. A serum is technically classified as positive when the OD at the 1/40 dilution is > 0.2. Each positive serum has a titre corresponding to the highest dilution at which it remains positive. However, on the basis of practical experience, positive results of 1/40–1/80 should be considered within the doubtful range.

## Results

### Experimental vaccination and serological profiles

#### Experiments conducted in Australia

##### Vaccination of laboratory-bred wild rabbits

**Figure 1:**
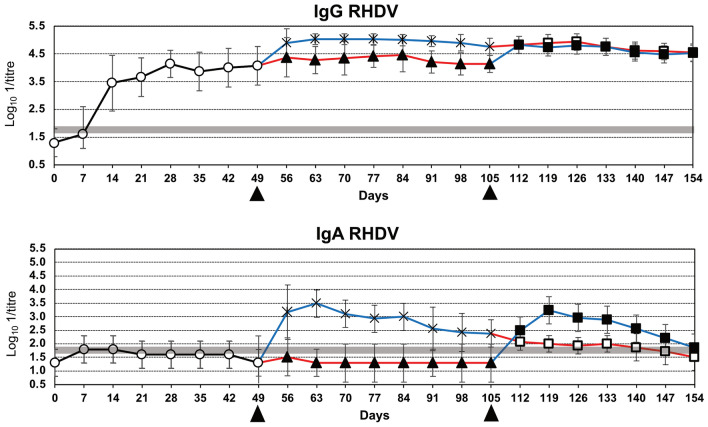
**IgG and IgA ELISA results of vaccinated and challenged wild rabbits**. Mean Log_10_ antibody titres following initial RHD vaccination and subsequent double challenge with RHDV. The blue line indicates the group of rabbits subjected to oral challenge, and the red line indicates the group of rabbits subjected to subcutaneous challenge. **O** indicates antibody titres after vaccination, *N* = 10. Antibodies titre after the first RHDV challenge: **X** rabbits, *N* = 5; ▲ rabbits, *N* = 5. Antibodies titre after the second RHDV challenge: □ rabbits, *N* = 5 (rabbits randomly selected: 3 from ▲ and 2 from **X**); ■ rabbits, *N* = 5 (rabbits randomly selected: 2 from ▲ and 3 from **X**). The black arrow on the *x*-axis indicates the time of the two consecutive challenges. The values within the grey bar are uncertain or indicate the presence of traces of antibodies. The error bars around each mean value are standard deviations; titres of orally and subcutaneously treated rabbits differ significantly where the error bars do not overlap.

Before vaccination, serum testing confirmed that all ten rabbits were seronegative for all RHD antibody isotypes, IgA, IgG and IgM. After vaccination, as expected, all rabbits exhibited a transient IgM response with an average titre of 1/10240 (1/Log 4.01) at 10 days, which declined to zero within 2 months (data not shown). This was followed by a trace IgA response in some rabbits and, subsequently, by an increased IgG response (Figure 1). In fact, by Day 7 after vaccination, positive IgA titres (just above 1/80–1/Log 1.9) were observed in five rabbits, while minimal reactivity was detected in the remaining five. These low IgA levels were recorded in three rabbits until day 49, after which the IgA in most rabbits’ serum disappeared completely. By Day 49, only two rabbits still had measurable IgA titres around 1/80 (1/Log 1.9).

Three rabbits showed initial traces of IgG within 7 days of vaccination. Antibody responses were highly variable, and at 14 days, IgG titres ranged from 1/160 (1/Log 2.2) to slightly above 1/10240 (1/Log 4.01) (mean titre = 1/5200 1/Log 3.71). The maximum titres observed, estimated to be around 1/20,480 (1/Log 4.31), were recorded in three rabbits at 28 days. At 49 days, IgG titres varied between 1/1280 (1/Log 3.11) and 1/20,480 (1/Log 4.31) (mean titre = approximately 1/10,240–1/Log 4.01). The changes in IgG titres were confirmed as significant by analysis of the log-transformed data (ANOVA, F (7,72) = 128.100, *P* < 0.001), indicating a steady increase in titres from Day 0 to Day 28 and then stabilisation.

##### Initial exposure to live RHDV (first challenge)

On Day 49 post-vaccination, all rabbits received 0.5 mL of RHDV preparation either orally (*N* = 5) or via subcutaneous inoculation (*N* = 5). None subsequently exhibited signs of disease; however, their serological responses are shown in Figure 1. In addition, none of the challenged rabbits exhibited a detectable IgM response (data not shown). The absence of a primary challenge response indicated that vaccination was effective, meaning no new naïve B lymphocytes had been activated to produce IgM.

Orally dosed rabbits exhibited significant increases in IgA titres from 7 days post-challenge. By 14 days post-challenge, titres ranged from 1/1280 (1/Log 3.11) to 1/5120 (1/Log 3.71), but then declined (ANOVA, F (11,48) = 23.130, *P* < 0.0001). In contrast, three of five rabbits in the subcutaneously inoculated group showed only a slight, non-significant rise in titre over their pre-challenge IgA levels at 7 days post-challenge; one remained unchanged, and one rabbit tested negative by the IgA test. By 14 days post-challenge, the average IgA titre remained at trace levels; analysis of log-transformed data indicated that titres did not differ from the post-vaccination levels prior to challenge (ANOVA, F (11,48) = 0.467, *P* = 0.914). This demonstrated that the significantly higher IgA titres were associated with the oral route of inoculation (ANCOVA, F (1,87) = 100.190, *P* < 0.0001).

By Day 7 post-challenge, the IgG antibody titres of the five rabbits dosed orally had increased significantly, with four of them showing titres over 1/40960 (1/Log 4.61), while the fifth had risen from around 1/5120 (1/Log 3.71) to approximately 1/10240 (1/Log 4.31). By Day 14, IgG titres in all rabbits exceeded 1/40960 (1/Log 4.61). In the group inoculated subcutaneously, IgG titres at 7 days post-RHDV rose above pre-existing levels in only two rabbits, while the titres of the remaining three remained unchanged. By Day 14, titres had decreased slightly in the two rabbits with initially high levels, but remained roughly the same in the other three. Analysis of log-transformed data at 14 days showed no significant increase in IgG titres above pre-challenge levels (ANOVA, F (8,36) = 0.470, *P* = 0.869). Once again, the route of inoculation was linked to IgG antibody titres (ANCOVA, F (1,87) = 51.681, *P* < 0.0001).

##### Re-exposure to live RHDV (second challenge)

Rabbits were re-exposed to RHDV 8 weeks after the initial challenge, on Day 104 post-vaccination (Figure 1). Again, significant IgA responses were observed following oral inoculation, closely paralleling results obtained following the first challenge. Furthermore, the average IgA titre in the subcutaneously inoculated rabbits, which was initially about 1/160 (1/Log 2.2), steadily fell to become negative over the remaining 7 weeks. This indicated that no new IgA had been produced and antibody loss had continued through natural waning. On the basis of both experiments, the oral route of challenge consistently elicited significant IgA responses, and that result was apparent even among rabbits with different inoculation histories.

**Figure 2:**
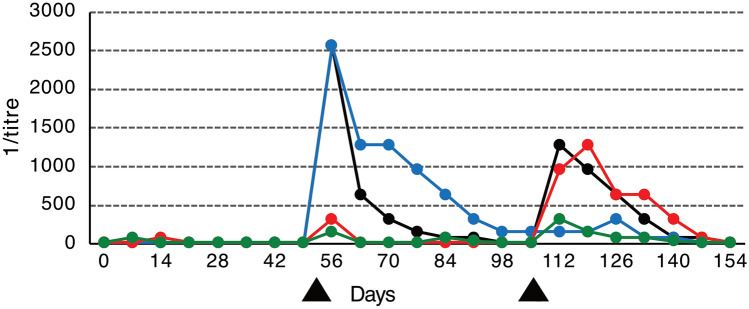
**IgA ELISA results from individual wild rabbits challenged via different routes.** Serum IgA titres in four individual rabbits following initial RHD vaccination and subsequent double challenge with RHDV using the alternative route [oral (Oc) or subcutaneous (Sc)]. The black line represents Oc + Oc, the blue line Oc + Sc, the red line Sc + Oc, and the green line Sc + Sc. The black triangle indicates the days of challenge. For illustration purposes, differences in responses to routes of challenge are more clearly demonstrated using reciprocal titres rather than log titres as used in statistical analyses to normalise observed variation between rabbits.

The data we obtained from individual rabbits during the two experiments in Australia were important for determining the magnitude of changes in IgA titres that might be considered indicative of re-exposure to live RHDV among wild rabbits in the field. Figure 2 gives examples which suggest that an increase in IgA titres exceeding two ELISA dilutions in successive samples or an IgA titre in an individual serum sample of 1/1280 (1/Log 3.1) or more would be useful criteria.

#### Experiments conducted in Italy

##### Testing of homogenates

Both formalin-treated and non-formalin-treated homogenates demonstrated very similar reactivity in the sandwich ELISA, with an endpoint titre of roughly 1/1000 (data not shown). None of the three rabbits inoculated orally with the inactivated homogenate showed clinical signs of RHD, and all remained seronegative in the RHDV cELISA 2 weeks after inoculation.

##### Serological results after vaccination and challenge

**Figure 3:**
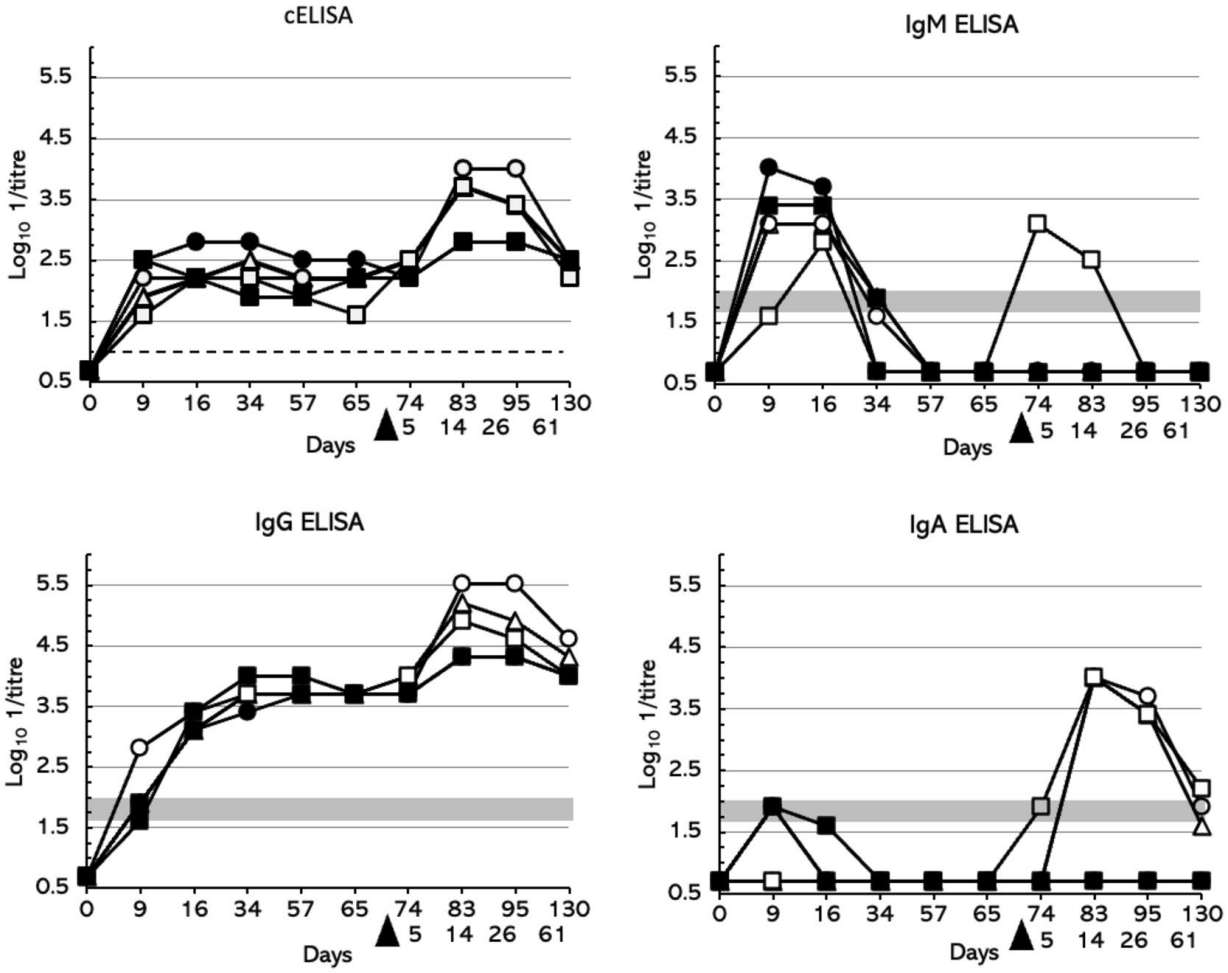
**ELISA results for vaccinated and challenged farm rabbits.** Each of the five symbols used in the graph corresponds to a single rabbit. The three empty symbols (□, △, ○) correspond to rabbits challenged with the live virus, while the black ones (■, ●) correspond to those challenged with the inactivated virus. The arrow below the *x*-axis indicates the day of the challenge. The numbers on the *x*-axis correspond to the days after vaccination, while those below correspond to the days after challenge. The values within the grey bar are uncertain or indicate the presence of traces of antibodies.

The ELISA test results for RHD vaccination are shown in Figure 3 and align with existing knowledge of RHD humoral immunity in rabbits. All rabbits tested seropositive by cELISA at 9 days post-vaccination, with titres ranging from 1/40 (1/Log 1.6) to 1/320 (1/Log 2.51). During this early period, IgM remained the dominant antibody class; however, within a week, the IgG response began and quickly became dominant. Conversely, by Day 9, three of five rabbits exhibited a doubtful presence of IgA (1/80–1/Log 1.9), and by Day 16, only one rabbit still showed possible traces. At 2 months post-vaccination, all rabbits were positive only for IgG, with similar titres around 1/5120 (1/Log 3.71) in the IgG ELISA, corresponding to 1/160 (1/Log 2.2) in cELISA. Five days after the challenge, the serological profiles of the rabbits started to change depending on the type of liver homogenate used. Interestingly, while the IgA titre of the three rabbits challenged with the live virus increased from a negative value to 1/10240 (1/Log 4.01) over approximately 2 weeks and then decreased to a minimal level within about 7 weeks, the rabbits receiving the formalin-inactivated homogenate did not develop an IgA response. Similarly, in the three rabbits treated with the live virus, IgG titres increased by approximately 16-fold and then returned to initial levels within about 5 weeks; in contrast, the inactivated virus caused only a modest increase of 2–4 times in titres. The IgM profiles varied. Regardless of the challenge homogenate type, four out of five rabbits tested negative in the IgM ELISA, while one showed an increase 5 days post-challenge, peaking at 1/1280 (1/Log 3.11) before rapidly declining. Notably, this rabbit exhibited a lower IgM response after vaccination compared with the titres seen in the others.

Finally, the variation in the total Ig profile confirmed the above results. In fact, the cELISA titres rose about 16-fold roughly 2 weeks after live virus challenge, reaching levels similar to those of RHD convalescent rabbits, but returned close to the previous level of 1/160 (1/Log 2.2) within 7 weeks.

#### Serological survey in the ANCI rabbit farm

##### Serological survey of does that received multiple vaccinations

**Figure 4:**
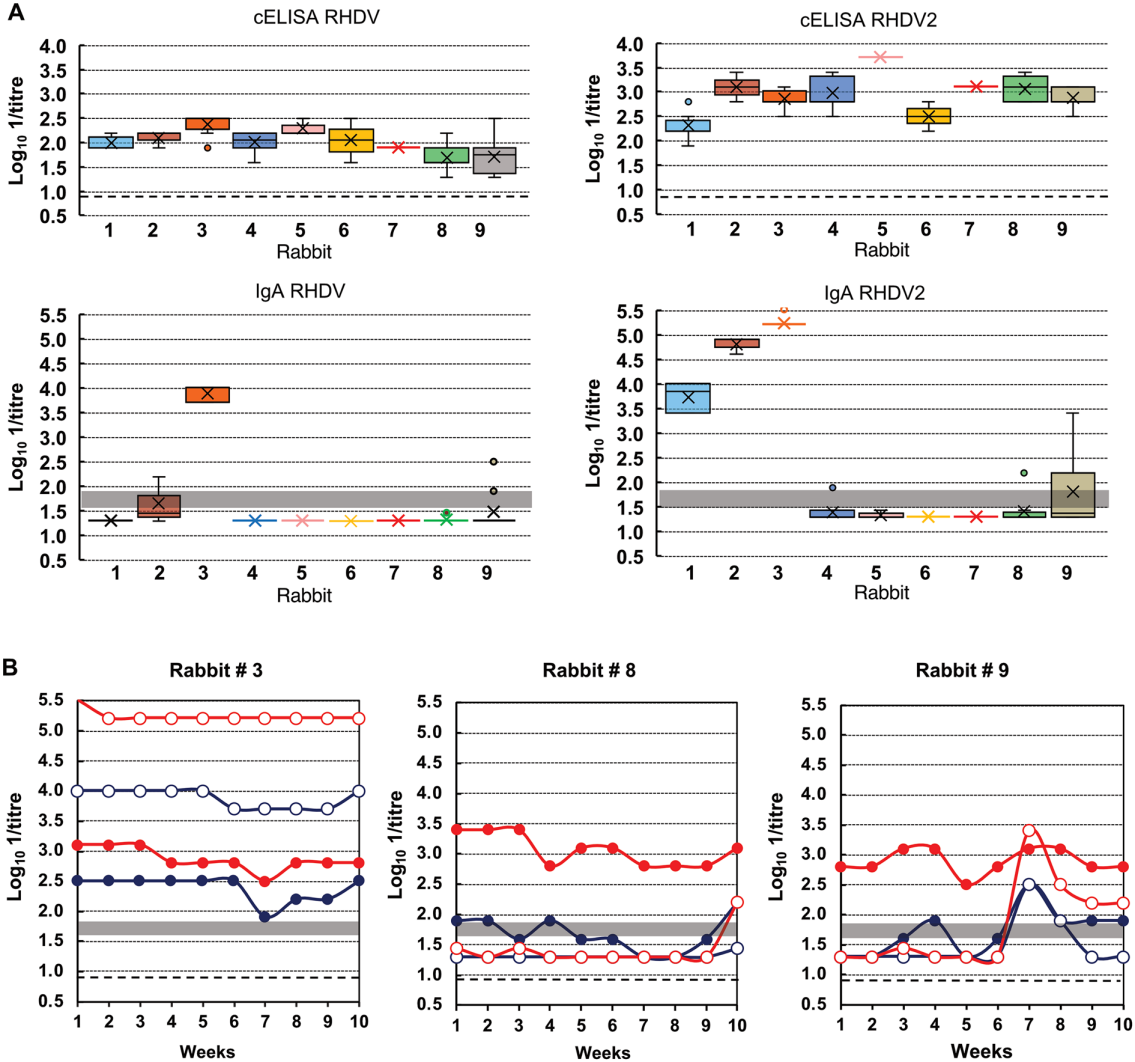
**ELISAs results of the nine females listed in Table **1** and sampled weekly**. Panel **A** The box plots (including the median) summarise the serological results from mother 1 to mother 9 listed in Table 1. Each box contains all the samples collected for each mother during the study. The boxes are coloured to make it easier for the reader to compare the results of the four different ELISA tests. The *y*-axis values represent the Log_10_ of the inverse of ELISA titres. Panel **B** The numbers on the *x*-axis indicate the ten consecutive weeks of sampling. The red curves represent RHDV2 results, while the blue curves represent RHDV results. Filled circles represent cELISA results, whereas empty circles indicate IgA. The dashed line at Log 0.7 (negative sera, titre < 1/10) marks the cELISA cut-off. Values within the grey bar are uncertain or trace amounts of IgA. Clinical cases of RHD caused by RHDVa appeared on the farm around the fifth week of the study.

Table 1 presents the main anamnestic data for the tested females, while Figure 4 displays the overall serological results graphically. Clearly, all females had previously received two doses of an RHDV2 vaccine and tested positive for RHDV2 by cELISA, with titres ranging from 1/80 (1/Log 1.9) to 1/5120 (1/Log 3.71). All sera also tested positive for RHDV cELISA, but almost invariably with titres 4–32 times lower than those for RHDV2.

**Table 1:** **Summary of the available anamnestic data of the mothers**

No	Age at R2	Age at Ra	IgA2	23 June 2014	07 July 2014	25 August 2014
1	566	1070	1/6500	p		p
2^*^	376	880	1/81920		p	
3	−28	476	1/163,840	p		p
4	−76	428	N	p		p
5^*^	−90	414	N	p		
6^*^	−90	414	N		p	
7	−97	407	N—1/160	p		p
8^*^	−97	407	N		p	
9	−220	284	N—1/2560	p		p

“Age at R2” and “Age at Ra” refer to the age in days of the rabbits at the time of RHD outbreaks caused by RHDV2 and RHDVa, respectively. The negative sign (“−”) indicates the number of days after the RHDV2 outbreak when the rabbits were born. “IgA2” represents the average IgA titre of the 10 or 3 [number with asterisk (*)] samples. “N” indicates rabbits that tested negative in ELISA. “p” signifies the dates on which each doe gave birth.

All tested sera were negative for both RHDV and RHDV2 IgM (data not shown). Regarding IgA, three out of nine samples (#1, #2, #3) tested positive with high titres (from 1/5120–1/Log 3.71 to 1/163 840–1/Log 5.21) for IgA-RHDV2. Sample #9 remained negative until week 6 and then turned positive in the following weeks. The other five females remained negative throughout the survey period, although #22 showed an increase in IgA-RHDV2 with a titre just above the cut-off at the last sampling. Looking at the graphs (Figure 4), it is worth noting the variability in the ratio of anti-RHDV and anti-RHDV2 IgA titres in the three IgA-positive sera. In fact, while the IgA-RHDV titre was negative or with trace amounts in sera #1 and #2, it was clearly positive in serum #3, the one with the highest IgA-RHDV2 titre, although it remained about 20 times lower than that. However, the lowest IgA2/IgA ratio, i.e., approximately 6, was found in rabbit #9, in which the IgA seroconversion occurred at week 7, right during the RHDVa epidemic. Finally, it is worth noting that, in females testing positive, the IgA RHDV2 ELISA titre remained relatively stable over the 10-week study period.

Reviewing the anamnestic data in Table 1, no link was found between IgA positivity and the physiological state of the females (parturition). Likewise, since all nine females received the same vaccination protocol, no link was observed between multiple vaccinations in rabbits and IgA induction. Conversely, an association emerged with the presence of rabbits on the farm during the previous devastating RHDV2 epidemic, which occurred in spring 2013. In fact, rabbits #1 and #2 had already been on the farm for over a year at the time of the RHDV2 epidemic, and #3 was born during the epidemic, while the remaining six rabbits that tested negative for IgA were born 2–3 months later (Table 1). A different situation applies to rabbit #9, whose seroconversion to IgA appears to be linked to an RHDVa infection.

##### Serological survey on young unvaccinated rabbits

**Figure 5:**
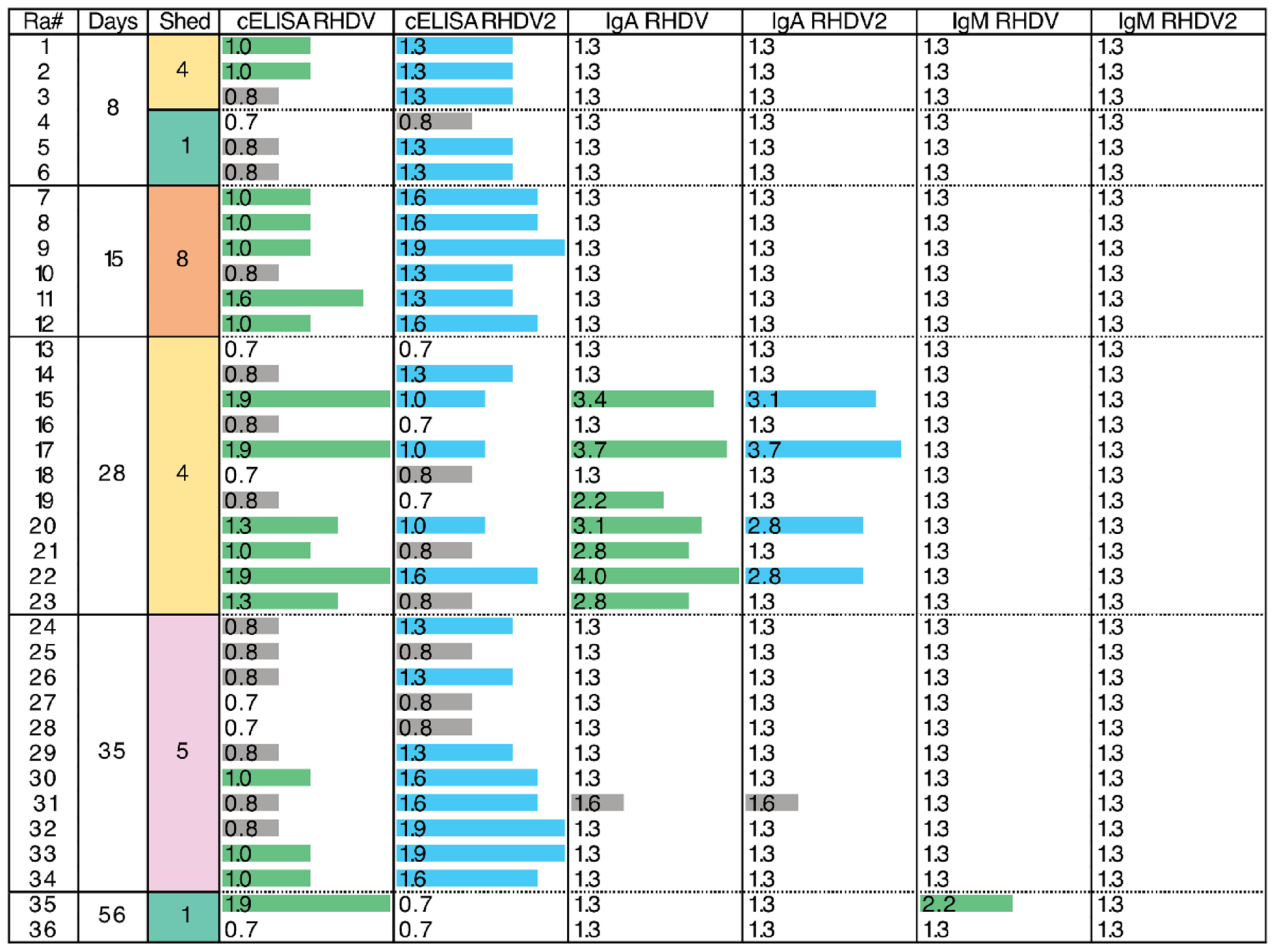
**ELISA results for unvaccinated young rabbits. ** “Days” indicates the approximate age of the rabbits at the start of the outbreak. “Shed” refers to the place where rabbits lived within the farm. ELISA results are reported as Log_10_ 1/titre and also represented as bars proportional to titre. Green bars refer to RHDV results and blue bars to RHDV2 results. Uncoloured cells correspond to negative ELISA results and grey cells to inconclusive results (possible trace of antibodies). Ra#, rabbit number.

At the start of the RHD outbreak, the first 12 rabbits shown in Figure 5 were still in the pre-weaning (lactation) phase, and cELISA test results indicated a clear presence of RHDV2 antibodies inherited from their mothers, despite all having received vaccinations with both RHDV/RHDVa and RHDV2 vaccines. In fact, RHDV2 cELISA titres were 2–8 times higher than RHDV cELISA titres, except for rabbit #11, which had a twofold higher RHDV titre. Conversely, 63% of the newly weaned rabbits, #13–#23, all housed in shed #4, showed an RHDV cELISA value 2–8 times higher than RHDV2, with IgA titres ranging from 1/160 (1/Log 2.2) to 1/5120 (1/Log 3.71). Surprisingly, all these rabbits also tested negative for IgM, indicating that the infection likely occurred weeks before sampling. For the rabbits in shed #5, #24–#34, although they belonged to the same category as those in shed #4, there was no evidence of IgA or of infection, with half of the rabbits losing their maternal antibodies over time. Finally, rabbit #35 in shed #1 displayed a titre of 1/80 (1/Log 1.9) in the cELISA test for RHDV and tested positive for RHDV IgM with a very low titre, but was negative for IgA. This profile is unique among the 36 rabbits, and it is indeed rather difficult to explain its origin.

##### Serological survey on young vaccinated rabbits

**Figure 6:**
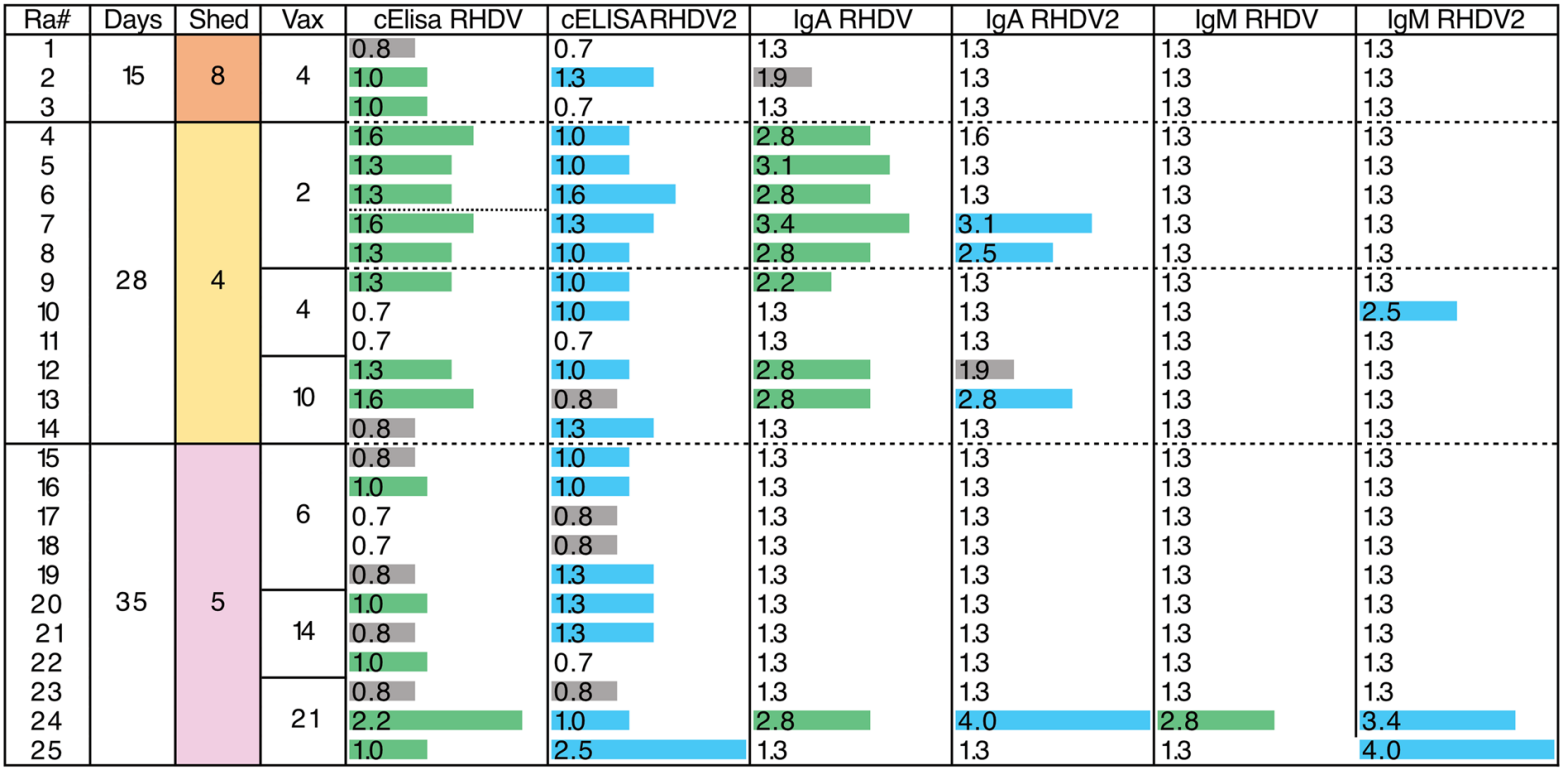
**ELISA results of first-time vaccinated rabbits.** “Days” indicates the approximate age of the rabbits at the start of the outbreak. “Shed” refers to the place where rabbits live within the farm. “Vax” indicates how many days after vaccination blood was taken from the rabbits. ELISA results are reported as Log_10_ 1/titre and also represented as bars proportional to titre. Green bars refer to RHDV results and blue bars to RHDV2 results. Uncoloured cells correspond to negative ELISA results and grey cells to inconclusive results (possible trace of antibodies). Ra#, rabbit number.

Although all the rabbits shown in Figure 6 had been vaccinated for the first time by emergency following the outbreak, it must be considered that some of them were sampled only 2–6 days after vaccination, a period that is usually not sufficient to induce the first circulating IgM. This applies to three rabbits from shed #8 (#1, #2 and #3), eight from shed #4 (#4–#11) and five from shed #5 (#15–#19). The three rabbits in shed #8 displayed serological profiles with very low antibody levels, likely of maternal origin. In contrast, the serological profile of 72% of the rabbits housed in shed #4 (#4–#9, #12 and #13) confirmed the circulation of RHDVa within this shed. These rabbits showed medium-to-high IgA titres and a two- to eightfold dominance of RHDV antibodies over RHDV2 in cELISA. Notably, as with the unvaccinated rabbits, all tested negative for IgM except rabbit #10, which exhibited a very low level (1/320–1/Log 2.51) of IgM but was RHDV2-specific. In shed #5, while the first nine rabbits (#15–#23) still possessed maternal antibodies or tested negative, the last two rabbits (#24 and #25), sampled 21 days after vaccination along with #23, displayed distinct serological profiles. Rabbit #25 showed a classic post-vaccination profile, with an IgM RHDV2 titre of 1/10240 (1/Log 4.01) and an RHDV2 cELISA titre of 1/320 (1/Log 2.51), which is 32 times higher than the RHDV cELISA titre. The profile of rabbit #24 was more complex. Although its RHDV2 IgM titre of 1/2560 (1/Log 3.41) indicates a definite vaccine-induced antibody response, similar to serum #25, the dominant RHDV cELISA titre of 1/160 (1/Log 2.2) compared with 1/10 for RHDV2 suggests an RHDVa infection, further supported by elevated IgA levels, which are absent in rabbit #25. The vaccine failure observed in rabbits #17, #18 and #23 (only possible trace of antibodies), all sampled at least 2 weeks post-vaccination, may be attributed to circulating maternal antibodies that can suppress or delay vaccine efficacy, or to possible vaccination errors, which are plausible during an emergency situation.

## Discussion

Serology is recognised as a fundamental tool for diagnosing viral diseases and conducting epidemiological research. Its effectiveness largely depends on the understanding the interactions and relationships between each viral species and the host’s adaptive immune system. In this context, examining the trend of IgA production in response to infections and vaccinations is highly relevant, given IgA’s essential role in mucosal immunity and the fact that mucosal surfaces are the primary entry points for most viruses, including RHDV [18, 19].

In experimental trials conducted in Australia and Italy, approximately half of the rabbits developed serum IgA after vaccination; however, it was present at trace levels and lasted only a few weeks, in stark contrast to the high levels observed in convalescent RHD rabbits. The same outcome—virtually no relevant IgA level in serum—was seen in farmed animals vaccinated multiple times, conclusively confirming field diagnostic results indicating very low IgA levels to zero IgA in serum after vaccination. The large difference in serum IgA levels between vaccinated and convalescent rabbits results from several factors, including (i) a different “priming” site of the immune system, i.e., subcutaneous or intramuscular injections for the vaccine and mucosal sites in cases of infection and (ii) the status of the virus, whether inactivated or live.

Furthermore, the high IgA and, especially, IgG antibody responses found in the few convalescent rabbits that survived RHD are mainly due to very high viral replication in the liver. Then, this enormous amount of immunogen is rapidly transported in the bloodstream to other sites of adaptive system activation, such as the spleen and lymph nodes. In fact, when RHDV replication is restricted to the gut, the humoral response is also reduced by an average of 10–20 times. This occurs in young rabbits naturally resistant to RHD with very limited replication in the liver or in rabbits of all ages infected with non-pathogenic lagoviruses (RCVs), which are essentially enteric viruses [12, 13, 26, 28].

Although only traces of IgA are present in serum, the evidence that oral challenge with live RHDV induces high levels of IgA but not IgM, except in a single rabbit, suggests that IgA is produced directly by B lymphocytes already specifically primed by the vaccine. This idea is also supported by the finding of one rabbit with a significantly lower level of IgM after vaccination, which produced, in addition to IgA, a new wave of IgM after oral challenge, presumably as a result of limited priming from the vaccine immunisation.

Furthermore, in rabbits passively protected by inoculation with different amounts of specific IgG, and thus with an immune system not yet primed, the oral challenge with the live virus induced high levels of both IgM and IgA [15]. We did not explicitly measure IgA levels on mucosal surfaces, but their low serum levels and the apparent extensive viral replication after oral challenge suggest that the vaccination primes the mucosal immune system without generating a significant amount of mucosal sIgA (see also below). Overall, our results align with previous findings, suggesting that systemically administered vaccines can also trigger an immune response at the mucosal level [44]. The detailed mechanisms of this induction are poorly understood. However, they are generally believed to involve the migration of specialised immune cells, including antigen-presenting cells, from the vaccination site—where selection and development occur—to the mucosa-associated lymphoid tissue (MALT). Nevertheless, active viral replication is necessary to stimulate these cells to produce IgA, as oral inoculation with the inactivated virus did not.

A live virus challenge in rabbits led to an increase in serum IgA levels only when administered orally, not when given subcutaneously. Since IgG levels did not increase either, the most plausible explanation is that the vaccine-induced IgG promptly neutralises and promotes the clearance of subcutaneously inoculated RHDV, thereby preventing the activation of memory cells within the adaptive immune system.

Regarding the evidence that vaccination influences mucosal immunity, the consistent difference in titres we observed after vaccination with the commercial RHDV/RHDVa vaccine and the RHDV2 autovaccine is noteworthy. The higher titres in cELISA RHDV2, ranging from 4 to 16 times higher than in cELISA RHDV, are probably due to differences in immunogen concentration, which is higher in the autovaccine than in the commercial vaccines, despite both vaccines using the same adjuvant. Naturally, both vaccines offer protection against RHD, but it may be worth exploring whether they elicit different levels of mucosal immune stimulation. Indeed, the high serum titre induced by the autovaccine in mothers ensures a more sustained level of protection in young rabbits, which is especially important in cases of RHDV2 infection, as RHDV2 can cause RHD even in young rabbits. Of course, the induction of higher titres through vaccination could also be improved by using more effective adjuvants.

Several previous serological studies in wild rabbits, primarily in Australia, have shown a close association between rabbit RHDV infection or reinfection and serum IgA positivity [17, 45]. The main route of infection is oral, whether spread from rabbit to rabbit or via faeces or regurgitation of flies that feed on carcasses [9]. Additionally, the same association was observed in farmed rabbits during RHDV outbreaks, but with some surprising results. For example, breeding females showed high IgA levels despite no evidence of RHDV circulation on the farm, as indicated by the lack of increased RHD mortality in unvaccinated fattening rabbits. To clarify this, we collected and analysed sera from a group of nine multi-vaccinated females weekly. In 33% of them, we observed significant IgA levels that remained relatively stable throughout the entire observation period. Interestingly, using available anamnestic data, a clear correlation emerged between the presence of IgA and the presence of these rabbits during a devastating RHDV2 outbreak on the farm about 1.5 years earlier. The remaining rabbits, born 2–3 months after that outbreak, were all IgA negative, even when treated with the same vaccination protocol as the positive ones. This conclusively shows that repeated vaccination does not induce serum IgA.

The realisation that vaccination stimulates IgA production in rabbits previously infected with RHD shows a “reverse” mechanism compared with the one described earlier: in this case, specific cells of the adaptive immune system that were selected during RHDV infection at the mucosal level are boosted by the vaccine. This boost likely occurs through migration of these cells to the systemic immune sites, where the vaccine exerts its effect. It is important to note that similar outcomes have been observed in human studies. For example, in poliovirus, the induction of mucosal immunity—including IgA in both serum and saliva—by the inactivated vaccine depends on prior mucosal exposure to the live virus [46, 47]. Likewise, similar results were obtained when an adjuvanted bivalent norovirus virus-like particle (VLPS) vaccine was administered intramuscularly to previously infected individuals, eliciting the activation of IgA memory B cells with mucosal-homing characteristics [48, 49].

It should be noted that at least one of the nine breeding females experienced a temporary IgA spike, showing high titres for RHDV2 IgA and medium levels for RHDV IgA, coinciding with the start of RHD on the farm, an event that was clearly unforeseeable at the beginning of the investigation. An increase in IgA titres was also observed in about 70% of the sera from young rabbits in shed #4 collected 2–3 weeks after the RHD outbreak began. In both cases, the presence of IgA clearly indicates RHDVa infection, but the absence of specific IgM in all young rabbits raises some intriguing questions and observations. These rabbits were about 1 month old during the outbreak, so they were not yet susceptible to lethal RHD caused by RHDVa. Additionally, their serum tested positive for maternal antibodies, mainly anti-RHDV2, but also RHDV/RHDVa. Since their adaptive immune system was still not primed, if an infection occurred, they would have produced IgM, which would have remained present for at least a few weeks. Therefore, the complete absence of IgM, compared with an intermediate cELISA RHDV titre and a medium-to-high IgA RHDV titre in rabbits from shed #4, raises questions about the origin of these antibodies and when the infection likely occurred in this shed. The evidence that specific IgM antibodies have a short lifespan in cases of the non-pathogenic RCV infection at the gut mucosal level [28] supports the hypothesis of direct infection of young rabbits, probably occurring shortly before the formal start date of the outbreak. In fact, the alternative hypothesis of RHDVa infection in females, who would then have transmitted antibodies to their offspring, is unlikely, as it would shift the virus’s entry into the farm to well over a month before the onset of RHD in shed #7.

The information from our study on the relationship between RHDV infection and the adaptive immune system response provides considerations at the epidemiological and serological diagnostic levels.

The finding that RHDV is readily neutralised when injected subcutaneously into vaccinated rabbits suggests that the likelihood of virus transmission by biting insects in wild rabbit populations [9] is certainly lower when herd immunity is high, as in the case of recent RHDV/RHDV2 outbreaks. However, this route of transmission could still be relevant for young rabbits that lose maternal antibodies, particularly for RHDV2, which induces RHD in them.

The presence of serum IgA is a clear indicator of recent infection or reinfection, especially in wild rabbits that are not subject to vaccination campaigns. Therefore, the IgA test is a highly valuable tool for epidemiological studies, particularly in populations with high seropositivity rates, where clinical cases of RHD (evidence of disease) are rare and mainly observed in young individuals, and therefore limited to the reproductive season.

For farmed rabbits, IgA test results should be combined with each rabbit’s anamnestic data, considering only those born at least 2–3 months after the last RHDV outbreak as newly infected with RHD. Therefore, the IgA test can be used to detect new infections and assess ongoing viral circulation on a farm after extensive emergency vaccination. It becomes an effective tool in the control and eradication strategy to end an outbreak and confirm a farm’s RHD-free status.

## Data Availability

The datasets used during the current study are available from the corresponding author upon reasonable request.
